# Early Effects of Treatment Low-Dose Atorvastatin on Markers of Insulin Resistance and Inflammation in Patients with Myocardial Infarction

**DOI:** 10.3389/fphar.2016.00324

**Published:** 2016-09-26

**Authors:** Olga Gruzdeva, Evgenya Uchasova, Yulia Dyleva, Olga Akbasheva, Victoria Karetnikova, Olga Barbarash

**Affiliations:** ^1^Federal State Budgetary Institution Research Institute for Complex Issues of Cardiovascular DiseaseKemerovo, Russia; ^2^State Budget Educational Institution of Higher Professional Education “Siberian State Medical University” of the Russian Federation Ministry of HealthTomsk, Russia

**Keywords:** statin, myocardial infarction, inflammation, adipokine, lipid

## Abstract

Dyslipidemia is one of the primary causes of cardiovascular disease. Therefore, attention has been focused on the development of drugs that normalize lipid levels and exert an effect on markers of atherothrombosis, insulin resistance (IR), and inflammation. Atorvastatin is a drug with not only lipid-lowering potential, but it has multiple non-lipid effects. This study aimed to evaluate atorvastatin effects on lipid, adipokine, IR, and inflammatory statuses in patients with myocardial infarction (MI) in an in-hospital setting. This study included 66 patients with confirmed ST-segment elevation MI, who were treated with atorvastatin 20 mg/day starting on day 1 of MI, without any dose changes. The comparison group consisted of 60 patients receiving standard anti-anginal and anti-thrombotic therapy. During the hospital stay, both groups showed a reduction in total cholesterol level and free fatty acids and increased concentrations of apolipoprotein A, especially those patients receiving atorvastatin. On day 1 of MI, patients in both groups had elevated levels of leptin by 2.9- to 3.3-fold, but the leptin levels decreased by 40.3% and were significantly lower than in patients not taking statins. The treatment with atorvastatin was associated with a decrease in C-reactive protein and interleukin-6 by 23.1 and 49.2%, respectively, compared with baseline values. In the group of patients on standard therapy, there was a decrease of interleukin-6 by 31.7%. Atorvastatin administered early on during hospitalization to patients with MI contributed to the improvement of lipid, adipokine and pro-inflammatory statuses and decreased IR.

## Introduction

Despite recent advances in treatment options aimed at reducing the rate of ischemic events in the first days following disease onset, patients with myocardial infarction (MI) remain at high risk of cardiovascular events. Approximately 38% of women and 25% of men will die within 1 year of a first recognized heart attack (Rosamond et al., 2008). These data suggest the need to develop clinically effective strategies to target pathophysiological mechanisms responsible for the development and course of MI.

Dyslipidemia is one of the major causes of MI that is considered a complex multifactorial disease. Recently, insulin resistance (IR) has been included as a potential risk factor for MI. Its development is associated with a diverse set of interrelated pathological mechanisms, namely atherothrombosis. These mechanisms involve the activation of local and progressive inflammatory processes in the atherosclerotic plaque concurrent with endothelial dysfunction, which subsequently contribute to the development of MI (Cefalu, 2001). Therefore, it is relevant to develop drugs that can simultaneously normalize the maximum number of elements involved in the pathogenesis of MI.

Taking into account that one of the major causes of cardiovascular diseases is high circulating and tissue cholesterol levels, drugs that not only improve impaired lipid transport, but also exert beneficial effects on markers of atherothrombosis, IR, and inflammation are necessary. One such promising drug is atorvastatin, which is known to reduce cholesterol levels by inhibiting hydroxymethylglutaryl-coenzyme A reductase. Its beneficial effects are associated not only with its lipid-lowering potential, but also with pleiotropic effects (improvement of endothelial function, reduction of aseptic inflammation, among others) according to results of long-term studies (Ichihara and Satoh, 2002; Aronov, 2004; Rodríguez-Calvo et al., 2009). Therefore, the study of the short-term effects of atorvastatin on metabolic and functional disorders during early stages of MI treatment could be useful to determine the prognosis and post-MI management in this group of patients. The aim of this study was to study the effects of atorvastatin on lipid parameters, leptin, adiponectin, IR markers, plasminogen activator inhibitor type 1 (PAI-1), and inflammatory parameters in patients with MI in an in-hospital setting.

## Methods

### Ethical considerations

The study protocol was approved by the Local Ethics Committee of the Federal State Budgetary Institution Research Institute for Complex Issues of Cardiovascular Diseases and was developed according to WMA Declaration of Helsinki on Ethical Principles for Medical Research Involving Human Subjects, 2000 edition, and the “GCP Principles in the Russian Federation” approved by the Russian Ministry of Health (19.06.2003). All patients gave written informed consent.

### Study design

This was an open-label, prospective, comparative, controlled, 12 day study performed in Kemerovo Cardiology Dispensary in 2010. The inclusion criteria were as follows: confirmed diagnosis of MI based on clinical, electrocardiographic, echocardiographic findings, and biochemical features of the disease (Thygesen et al., 2007). The exclusion criteria were as follows: statin therapy prior to MI; serious diseases affecting prognosis, such as renal failure, anemia, oncological diseases, hepatic failure, inflammatory, and infectious disease during exacerbation; autoimmune diseases; prolonged treatment with corticosteroids; and diabetes.

We included 66 patients with confirmed ST-segment elevation MI, who received atorvastatin (atomax, JSC “Makiz Pharma,” Russia) 20 mg/day starting on the first day of MI onset. The dosage of atorvastatin was not increased (Group 1). The study presents the results of observations during the period of 12 days from initial administration. The efficacy of atorvastatin was assessed on 65 patients owing to one treatment discontinuation. Therefore, the efficacy of atorvastatin therapy was assessed in 65 patients.

Sixty patients receiving standard anti-anginal and anti-thrombotic therapy were retrospectively selected using a case-control method from the ST-segment Elevation Acute Coronary Syndrome Registry (*n* = 423) in the Kemerovo Cardiology Dispensary between 2012 and 2013. These patients were included in the comparison group (Group 2). Patients in this group did not take statins during the pre-hospital or hospitalization periods.

The control group included 40 subjects (30 were male and 10 were female) aged 58 (56.3; 60.2) years, without cardiovascular and endocrine disease, who were comparable to MI patients in age and sex.

During the in-hospital period (mean period of 12 days), all the patients (Group 1) received β-blockers, ACE inhibitors, calcium channel blockers, diuretics, nitrates, aspirin, heparin, clopidogrel and statins. Patients group 2 received all recommended medications except statins.

### Assays

The serum of each patient was separated from venous blood by centrifugation at 3000 × g for 20 min and stored at −70°C. On days 1 and 12 after MI onset, serum glucose, total cholesterol (TC), triacylglycerol (TAG), free fatty acid (FFA), low-density lipoprotein cholesterol (LDL), very-low-density lipoprotein cholesterol, apolipoprotein B (apo-B), apolipoprotein A1 (apo-A1), and high-density lipoprotein cholesterol (HDL) levels were measured at the same study time-points using standard Thermo Fisher Scientific test systems (Thermo Fisher Scientific Oy, Vantaa, Finland) in a Konelab 30i biochemistry analyzer (Thermo Fisher Scientific Oy). C-peptide measured by ELISA with BioMedica (Sydney, Australia) and insulin levels Diagnostic Systems Laboratories (Webster, TX, USA) laboratory kits, respectively. The intra-assay coefficients of variation (CV) for insulin and C-peptide ELISA were 3.8 and 4.2%, respectively, and the inter-assay CVs were 6.9 and 7.9%, respectively. Adipokine (leptin, adiponectin) levels were measured using BioVendor assay kits (Brno, Czech Republic) and intra-assay CVs were 5.9 and 6.8%. Patient prothrombotic potential was assessed by determining PAI-1 levels, which were measured using Technoclone GmbH assay kits (Vienna, Austria). The intra-assay CVs were 4.9 and 5.8%.

Proinflammatory factors (interleukin-6, IL-6; eBioscience, Vienna, Austria) and C-reactive protein (CRP) (Biomerica, Irvine, CA, USA) were assessed using standard test kits (CV, 7.03–8.99%, and CV, 2.3-4.1%) Serum glucose, insulin, and C-peptide levels were measured to assess carbohydrate metabolism and to diagnose IR. The homeostasis model assessment of IR (HOMA-IR) index was calculated on days 1 and 12 after MI onset. A HOMA-IR value > 2.77 was established as the cut-off value indicating IR.

### Statistical analysis

Statistical analysis was performed using Statistica 6.1. software (InstallShield Software Corp., Chicago, IL, USA). Results are presented as median (Me) and 25 and 75% quartiles Me (Q1;Q3). Statistical analyses were performed using the nonparametric Mann–Whitney test for unpaired samples and the Wilcoxon test for paired samples. Spearman's correlation coefficient was calculated to analyze correlations between variables.

## Results

Atorvastatin was generally well-tolerated, except in one patient. In that case, the drug administration was discontinued because of the development of dyspepsia. The patient experienced nausea within a week of beginning treatment with atorvastatin.

The groups were well-matched for sex, age, and presence of cardiovascular risk factors, such as hypertension, smoking, and overweight. Over 41% of patients in both groups had a family history of coronary artery disease (Table 1). Chronic pyelonephritis and peptic ulcer disease prevailed among comorbidities. The activity of CPK-MB did not differ significantly in both groups [Group 1, 129.6 (111.4;135.6) U/L, Group 2, 146.3 (121.5;156.2) U/L, *p* = 0.942]. No clinical signs of congestive heart failure were observed in roughly 80% of patients in both groups, which was estimated using the Killip classification at the time of admission and during the follow-up period (Killip and Kimball, 1967). Over 40% of patients experienced arrhythmias and were diagnosed according to the generally accepted criteria (Kryzhanovsky, 2001). The parameters of the structural and functional states of the left ventricle did not differ significantly between the groups. The mean left ventricular ejection fraction was 53.5 (52.2;54.4)% in Group 1 and 45.2 (43.5;46.2)% in Group 2 (*p* = 0.385). Mean left ventricular end-diastolic volume was 153.2 (151.6;154.4) mL in Group 1 and 154.4 (151.3;16.9) mL in Group 2 (*p* = 0.768), whereas mean left ventricular end-systolic volume was 67.2 (65.4;69.5) in Group 1 and 74.2 (73.2;77.8) ± 7.8 mL in Group 2 (*p* = 0.14). Both groups were comparable for coronary atherosclerotic lesions: 14 (66.7%) patients in Group 1 and 10 (45.5%) patients in Group 2 had multivessel (more than three-vessel disease) coronary disease (*p* = 0.688) according to the results of coronary angiography performed at the time of admission. Percutaneous coronary intervention of the infarct-dependent artery was performed as reperfusion therapy in all patients.

**Table 1:** **Clinical and demographic data of the patient cohort, ***n*** (%)**.

**Variable**	**MI patients**		***P***
	**Group 1, *n* = 66**	**Group 2, *n* = 60**	
Men, *n* (%)	58 (87.8)	55 (91.6)	0.862
Age, years	60.4 (56.6;62,3)	56.5 (52,3;61,2)	0.118
**CAD RISK FACTORS**			
Arterial hypertension, *n* (%)	52 (78.7)	55 (91.6)	0.861
Current smoking, *n* (%)	45 (68.2)	50 (83.3)	0.854
Family history of ischemic heart disease, *n* (%)	30 (45.5)	29 (48.3)	0.911
BMI, kg/m^2^	26.8 (24.8;27.5)	27 (25.5;28.6)	0.445
**PRIOR MEDICAL HISTORY**			
Angina pectoris signs prior to MI onset	33 (50.0)	29 (48.3)	0.914
Prior MI	12 (18.2)	9 (15)	0.858
A positive history of acute cerebrovascular accidents/transient ischemic attacks	3 (4.6)	0	0.641
**COMORBIDITIES**			
Chronic bronchitis *n* (%)	6 (9.1)	5 (8.3)	0.786
Bronchial asthma, *n* (%)	3 (4.6)	5 (8.3)	0.641
Ulcerous disease in remission *n* (%)	3 (4.6)	5 (8.3)	0.786
Chronic pyelonephritis *n* (%)	9 (13.6)	12 (20)	0.858
**DEPTH OF LESION**			
**MI**			
- Q-wave	48 (72.7)	42 (70)	0.927
- non Q-wave	18 (27.3)	18 (30)	0.894
**MI localization**			
- Posterior;	36 (54.5)	28 (46.6)	0.833
- Posterior and RV;	14 (21.2)	13 (21.6)	0.99
- Anterior	16 (24.2)	19 (31.7)	0.781
- Circular	0	5 (8.3)	0.641
**POST-MI COMPLICATIONS (IN-HOSPITAL PERIOD)**			
**ACF (Killip)**			
I	54 (81.8)	46 (76.6)	0.99
II	8 (12.1)	7 (11.6)	0.99
III	4 (6.1)	7 (11.6)	0.786
IV	0	0	0.99
**Complications followed MI (in the in-hospital period)**			
- Arrhythmias	27 (40.9)	27 (45)	0.911
- Early post-infarction angina pectoris	12 (18.2)	15 (25)	0.762
**IN-HOSPITAL TREATMENT**			
- β-AB	65 (98.5)	60 (100)	0.936
- ACEs	57 (84.4)	49 (81.6)	0.932
- CCB	60 (90.9)	60 (100)	0.871
- Diuretics	24 (36.4)	28 (46.6)	0.818
- Nitrates	12 (18.2)	15 (25)	0.762
- Aspirin	63 (95.5)	60 (100)	0.936
- Heparin	63 (95.5)	60 (100)	0.936
- Clopidogrel	63 (95.5)	60 (100)	0.935

*MI, myocardial infarction; CAD, coronary artery disease; ACE, angiotensin-converting enzyme; CCB, calcium channel blockers; β-AB, beta blockers; ACEs, angiotensin-converting-enzyme inhibitor*.

The lipid profile showed significant changes in both groups at the time of hospital admission. These changes were commonly regarded as indicators of active atherogenetic process and risk for cardiovascular disease: elevated levels of total cholesterol, LDL-C, apo-B, apo-B/apo A1 ratio, TAG, and decreased levels of HDL cholesterol and Apo-A (Table 2).

**Table 2:** **Parameters of lipid transport function in blood and adipokine profile on days 1 and 12 after onset of MI**.

**Variable**	**Controls (*n* = 40)**	**Group 1**		**Group 2**	
		**Day 1**	**Day 12**	**Day 1**	**Day 12**
TC, mmol/L	4.5 (3.8;4.6)	5.80 (5.3;7.4)^a^	5.23 (4.86;5.42)^c^	5.59 (5.2;6.9)^a^	5.35 (4.96;6.52)
LDL, mmol/L	2.56 (2.1;2.76)	2.52 (2.2;3.01)^a^	2.36 (1.7;3.01)	2.54 (2.45;2.89)^a^	2.84 (2.75;3,53)^c^
HDL, mmol/L	1.26 (1.1;1.3)	0.88 (0.8;1.1)^a^	0.95 (0.77;1.13)	1.02 (0.98;1,12)^a^	1.11 (0,99;1,23)
VLDL, mmol/L	0.56 (0.4;0.9)	1.10 (0.9;1.4)^a^	1.03 (0.71;1.46)^a^	0.98 (0.95;1.12)^a^	1.26 (1.19;1.38)^c^
Apo-A, g/l	1.54 (1.3;1.65)	1.10 (0.9;1.3)^a^	1.23 (1.0;1.6)^a, b^	1.25 (1.12;1.35)^a^	1.50 (1.39;1.65)^c^
Apo-B, g/l	1.19 (1.0;1.5)	1.75 (1.2;1.9)^a^	1.43 (1.32;1.50)	1.40 (1.32;1.46)^a^	1.67 (1.58;1.76)^c^
TAG, mmol/L	1.20 (1.1;1.5)	2.17 (1.5;3.4)^a^	1.98 (1.85;2.01)	1.99 (1.87;2.23)^a^	2.34 (2.25;2.45)^c^
apo-B/apo-A1 ration	0.81 (0.7;1.0)	1.48 (0.9;1.8)^a^	0.92 (0.87;1.11)	1.24 (1.19;1.31)^a^	1.33 (1.28;1.40)^a, c^
FFA, mmol/L	0.35 (0.2;0.4)	1.92 (1.0;2.0)^a^	0.52 (0.49;0.59)^a, c^	1.88 (1.85;1.97)^a^	0.87 (0.78;0.98)^a, b, c^
Leptin, ng/ml	6.2 (5.6;7.3)	26.32 (24.9;27.9)^a^	15.71 (14.9;16.87)^a, c^	23.81 (21.89;24.76)^a^	19.33 (18.12;20.76)^a, b, c^
Adiponectin, ng/ml	12.1 (9.4;13.5)	13.12 (12.43;14.67)	15.06 (13.76;16.12)^a, c^	9.61 (9.01;10.98)	10.18 (9.89;10.74)

*TC, total cholesterol; TAG, triacylglycerol; HDL, high-density lipoprotein cholesterol; LDL, low-density lipoprotein cholesterol; VLDL, very-low-density lipoprotein cholesterol; apo-B, apolipoprotein B; apo-A1, apolipoprotein A*.

a Significant difference from control, (p < 0.05);

b Statistically significant differences in parameters between Group 1 and 2 on day 12, (p < 0.05);

c *Significant differences in parameters within one group on days 1 and 12, (p < 0.05)*.

Group 1 patients who received atorvastatin showed decreased total cholesterol levels and increased apo-A levels after 12 days of treatment. Group 2 patients, who did not receive statins, showed lower total cholesterol levels, but significantly high levels of LDL-C, apo-B, apo-B/apo A1 ratio, VLDL-C, and TAG.

Among the parameters of lipid metabolism, the most pronounced changes were observed in FFA levels (Table 2). On day 1 after MI, FFA levels in both groups showed a 9-fold increase compared with the control group. During the hospitalization period, FFA levels decreased in both groups, particularly in Group 1. In this group, FFA levels decreased by 83.3% compared with baseline values, whereas in Group 2, FFA levels were decreased by 53.5%. However, FFA levels were higher in both groups compared with those in the Control group (Table 2).

On day 1 of MI, patients had a 2.9- to 3.3-fold increase in leptin levels compared with the control group. Atorvastatin therapy (Group 1) resulted in a decrease in leptin levels by 40.3% and was significantly lower compared with the patients in Group 2 (Table 2). The assessment of the adiponectin/leptin ratio in patients with MI reported its significant decrease in all patients at the time of admission [mean values of 0.5 (0.48;0.52) to 0.38 (0.378;0.391) in Groups 1 and 2, respectively], compared with the ratio in the control group [mean values of 1.44 (1.43;1.448)]. The initiation of atorvastatin therapy increased the ratio values owing to the increased levels of adiponectin. However, this ratio remained at baseline values in patients who did not receive atorvastatin.

The results of the carbohydrate metabolism assessment are presented in Table 3. MI patients in both groups on day 1 showed a 1.5- to 1.6-fold increase in glucose levels, compared with the control group. Administration of therapies in both groups resulted in statistically significant reductions in glucose levels. Moreover, patients receiving atorvastatin presented with normal glucose levels, as those observed in healthy subjects (3.5–5.8 mmol/L). Further, beneficial effects of statins were demonstrated by C-peptide levels decreased by 37.6% compared with baseline values. C-peptide levels after treatment were significantly lower compared with those in patients who did not receive atorvastatin.

**Table 3:** **Markers of insulin resistance in patients with myocardial infarction on days 1 and 12 after MI onset**.

**Variable**	**Controls (*n* = 40)**	**Group 1**		**Group 2**	
		**Day 1**	**Day 12**	**Day 1**	**Day 12**
Insulin, mU/mL	11.6 (10.9;12,5)	12.55 (11.28;13.1)	10.81 (10,12;11.56)	12.73 (11.89;13.6)	11.79 (10.96;13,0)
C-peptide, ng/mL	1.4 (1.2;2.1)	1.8 (1.6;1.9)^a^	1.1 (0.8;1.9)^b, c^	1.61 (1.39;1,71)^a^	1.22 (1.12;1.34)^c^
Glucose, mmol/L	4.5 (4.1;5.2)	6.4 (5.9;6.8)^a^	5.48 (4.78;6.21)^c^	6.18 (5.89;7.94)^a^	5.6 (4,89;6.1)
HOMA-IR	2.6 (2.4;2.6)	3.3 (2.9;3.6)^a^	2.1 (2.2;3.1)^b, c^	3.71 (2.98;4.45)^a^	3.41 (2.67;4.56)^a^

*MI, myocardial infarction; HOMA-IR, homeostasis model assessment of insulin resistance*.

a Statistically significant differences with the control group, (p < 0.05);

b Statistically significant differences in parameters between Group 1 and 2 on day 12, (p < 0.05);

c *Significant differences in parameters within one group on days 1 and 12, (p < 0.05)*.

Despite the fact that insulin levels did not vary significantly during the follow-up period, the integral index of IR, HOMA-IR, had undergone significant changes. On day 1, HOMA-IR index increased 1.3-fold in Group 1 and 1.5-fold in Group 2 compared with the control group. The 12 day atorvastatin treatment showed a significant decrease in HOMA-IR by17.4% (2.1 [2.2;3.1]); its value decreased to normal. Group 2 patients who did not receive statins demonstrated elevated HOMA-IR, indicating the presence of moderate IR. PAI-1 levels in MI patients increased from 3.5- to 4.0-fold in comparison with the control group (Table 4). After 12-day atorvastatin treatment, PAI-1 levels significantly decreased by 35.3% compared with baseline values. PAI-1 levels remained 3.6 times higher in Group 2 patients, compared with the control group.

**Table 4:** **Levels of plasminogen activator inhibitor, interleukin 6, and C-reactive protein in patients with myocardial infarction on day 1 and 12 of hospitalization**.

**Variable**	**Controls (*n* = 40)**	**Group 1**		**Group 2**	
		**Day 1**	**Day 12**	**Day 1**	**Day 12**
Plasminogen activator inhibitor, ng/ml	35.3 (32.1;43.2)	125.9 (112.5;156.6)^a^	81.4 (75.6;92.5)^a, b, c^	144.61 (123.5;165.4)^a^	133.38 (123.6;154.2)^a^
C-reactive protein, mg/L	1.05 (0.8;1.5)	27.44 (26.3;30.8)^a^	20.88 (18.96;22.98)^a, c^	25.66 (24,31;26,87)^a^	24.27 (22.34;25.12)^a^
IL-6, pg/ml	3.90 (3.87;4.23)	22.66 (21.93;23.89)^a^	11.61 (10.87;12.56)^a, c^	20.35 (19.65;21.67)^a^	14.38 (13.56;15.45)^a, c, d^

a Statistically significant differences with the control group, (p < 0.05);

b Statistically significant differences in parameters between Group 1 and 2 on day 1, (p < 0.05);

c Statistically significant differences in parameters between Group 1 and 2 on day 12, (p < 0.05);

d *Significant differences in parameters within one group on days 1 and 12, (p < 0.05)*.

On day 1 of MI, CRP increased 23- to 25-fold, and IL-6 demonstrated a 6- to 7-fold increase compared with the control group (Table 4). Atorvastatin therapy reduced CRP levels by 23.1% and IL-6 levels by 49.2% in Group 1 compared with baseline values. However, patients receiving standard treatment reported a decrease in IL-6 levels by 31.7%.

## Discussion

Large prospective clinical studies have shown the positive effects of statin therapy on the long-term prognosis of patients with MI (Gotto et al., 2000; Pedersen et al., 2004); however, the effects of statins in an in-hospital setting in patients with MI are not clearly understood. A well-studied mechanism of statin activity is inhibition of HMG-CoA reductase, which enables the effective reduction of blood cholesterol levels (Bakker-Arkema et al., 1996; Rodríguez-Calvo et al., 2009). In our study, atorvastatin therapy, administered early on during the hospitalization of patients with MI, resulted in decreased total cholesterol levels.

According to the results of this study, the most pronounced change among the lipid metabolism parameters in MI patients receiving atorvastatin was the reduction of FFA levels, which could be explained by decreased FFA synthesis in the liver. Recent experimental studies reported that rats that were fed a fructose-rich diet showed elevated TAG levels and developed metabolic syndrome. Those treated with atorvastatin resulted in a decrease of FFA and TAG levels (Rodríguez-Calvo et al., 2009). The authors suggested that atorvastatin treatment blocked the activation of the carbohydrate response element binding protein (ChREBP), responsible for the synthesis of FFA from carbohydrates. Moreover, it is possible that atorvastatin treatment improves the use of FFA in the myocardial tissue of MI patients by peroxisome proliferator-activated receptors, which are involved in the cardiomyocyte energy metabolism (Rodríguez-Calvo et al., 2009).

Elevated FFA levels are now regarded as a marker of IR (Dresner et al., 1999). Thus, their reduction after atorvastatin therapy suggests a decrease in IR in patients with MI treated early on during hospitalization. This positive effect of the drug was confirmed by other changes in the parameters responsible for tissue sensitivity to insulin. We observed a significant decrease in the levels of C-peptide, serum glucose, and HOMA-IR after 12 days of atorvastatin treatment. Further, the values of the latter parameter were comparable to those in the control group. These results are consistent with those of other authors, confirming the beneficial effects of statins on peripheral tissue sensitivity to insulin and carbohydrate metabolism (Dresner et al., 1999).

In recent years, the roles of adipokines, leptin, and adiponectin have been actively discussed in the development of MI and its complications. Leptin and adiponectin are responsible for maintaining energy and metabolic homeostasis in physiological conditions. Normally, leptin protects the peripheral tissues from excessive accumulation of FFA, primarily by regulating feeding behavior in the hypothalamus. It is hypothesized that hyperleptinemia is concurrent with the development of IR, and thus, causes pathological changes in the cardiovascular system (Martin et al., 2008). Wallander et al. (2008) suggested that high leptin levels in the blood serum should be regarded as a predictor for recurrent cardiovascular events as well as for the development of carbohydrate intolerance in patients with MI. Unlike leptin, adiponectin plays a protective role, potentiating insulin effects on endothelial function, vessel wall tone, and platelet aggregation. The possible anti-atherogenic effect of adiponectin is currently under discussion, and its low level is seen as an indicator of IR, a high risk of developing metabolic syndrome and cardiovascular disease (Li et al., 2009). In this study, hyperleptinemia was found in all MI patients at the time of admission, and the subsequent 12 day therapy, especially in patients treated with atorvastatin, contributed to a marked reduction in plasma leptin levels. Furthermore, statin therapy showed a significant increase in adiponectin levels and the adiponectin/leptin ratio that can be regarded as a favorable effect of atorvastatin, which contributes by reducing the imbalance of adipokines and normalizing lipid metabolism in general.

Perhaps this is due to the effect of atorvastatin on balance adipoktine /fibroblast growth factor. As known, the functioning of adiponectin closely related fibroblast growth factor 21 (FGF21). Both hormones have multiple protective effects against cardiovascular diseases and metabolic dysfunction (Hui et al., 2016). Despite the fact that FGF21 and adiponectin have different structures and synthetic sources, these two hormones have striking functional similarity. In adipocytes transcription and secretion is induced adiponectin FGF21, which is partly dependent on the activity of PPARgamma. FGF21/adiponectin-axis protects from a variety of cardio-metabolic disorders through multiple organ mediating communication and is a promising target for therapeutic intervention in chronic diseases (Hui et al., 2016).

Current understanding of the pathogenesis of MI is based not only on the dyslipidemic theory and adipokine imbalance, there has been considerable emphasis on the activation of systems triggering inflammatory mechanisms and inducing disorders of the fibrinolytic system. Our results showed that levels of inflammatory markers, CRP and IL-6, were significantly higher in both groups of patients in the acute phase of MI, and in the early hospitalization period compared with the control group. It should be noted that atorvastatin treatment was accompanied by anti-inflammatory effects in the early in-hospital period, resulting in reduced CRP and IL-6 levels, which is also consistent with the results Gupta et al. (2008) who showed the efficacy of statin treatment within 4–6 weeks.

A significant reduction of PAI-1 levels in the hospitalization period was also determined after atorvastatin treatment. PAI-1 is considered to be a risk factor for atherothrombosis and MI, as well as a marker of IR, according to the results of current studies (Gotto et al., 2000). Alessi and Juhan-Vague (2006) suggested that obese patients with hyperinsulinemia and a genotype associated with increased transcription of PAI-1 were at high risk of developing MI. According to our findings, early effects of atorvastatin, manifested after 12 days of therapy, notably enhance the understanding of the pharmacological effects of statins and confirm their beneficial effects on the lipid metabolism after MI, thereby possibly preventing the development of further complications.

## Conclusion

The effects of treatment with atorvastatin 20 mg/day were shown during the early recovery period among patients with MI. The findings of the present study further confirm its pleiotropic effects. In addition to its lipid-lowering and anti-inflammatory effects, statin therapy reduced the severity of IR, based on reduced levels of FFA, IL-6, PAI-1, and improved adipokine status. All these parameters increase the efficacy of treatment in patients with MI and contribute to the improvement of their prognosis after hospital discharge.

## Limitations

Limitations of this study the outcomes are just biological index without clinical adverse events, which decrease its direction in clinical practice. And the short period of the observation also weaken the application and significance of this conclusion.

## Author contributions

OG was principal investigator, study co-ordinator and investigator, participated in all stages of recruitment of the patients and in analysis of the data, drafted, and reviewed critically the manuscript; OA, VK was study co-ordinator and investigator, participated in all stages of recruitment of the patients and in analysis of the data, drafted and reviewed critically the manuscript; EU and YD was study investigator, participated in all stages of recruitment of patients and reviewed critically the manuscript. OB was principal investigator. All other study investigators conducted the study and collected the data. All authors read and approved the final manuscript.

### Conflict of interest statement

The authors declare that the research was conducted in the absence of any commercial or financial relationships that could be construed as a potential conflict of interest.
